# *EGFR* Amplification and* IDH *Mutations in Glioblastoma Patients of the Northeast of Morocco

**DOI:** 10.1155/2017/8045859

**Published:** 2017-07-13

**Authors:** Nadia Senhaji, Sara Louati, Laila Chbani, Hind El Fatemi, Nawal Hammas, Karima Mikou, Mustapha Maaroufi, Mohammed Benzagmout, Said Boujraf, Sanae El Bardai, Marine Giry, Yannick Marie, Mohammed Chaoui El Faiz, Karima Mokhtari, Ahmed Idbaih, Afaf Amarti, Sanae Bennis

**Affiliations:** ^1^Laboratory of Bioactive Molecules: Structure and Functions, Faculty of Science and Technology of Fez, Sidi Mohamed Ben Abdellah University of Fez, Fez, Morocco; ^2^Pathological Anatomy and Molecular Pathology Service, University Hospital Hassan II of Fez, Fez, Morocco; ^3^Laboratory of Biomedical and Translational Research, Faculty of Medicine and Pharmacy of Fez, Sidi Mohamed Ben Abdellah University of Fez, Fez, Morocco; ^4^Department of Radiology, University Hospital Hassan II of Fez, Fez, Morocco; ^5^Department of Neurosurgery, University Hospital Hassan II of Fez, Fez, Morocco; ^6^Department of Biophysics and Clinical MRI Methods, Faculty of Medicine, Sidi Mohamed Ben Abdellah University of Fez, Fez, Morocco; ^7^The Clinical Neuroscience Laboratory, Faculty of Medicine, Sidi Mohamed Ben Abdellah University of Fez, Fez, Morocco; ^8^Inserm, U975, 75013 Paris, France; ^9^Institut du Cerveau et de la Moelle épinière, ICM, 75013 Paris, France; ^10^Inserm, U 1127, 75013 Paris, France; ^11^CNRS, UMR 7225, 75013 Paris, France; ^12^Sorbonne Universités, UPMC Univ Paris 06, UMR S 1127, 75013 Paris, France; ^13^AP-HP, Groupe Hospitalier Pitié-Salpêtrière, Laboratoire de Neuropathologie R. Escourolle, 75013 Paris, France; ^14^AP-HP, Hôpital de la Pitié-Salpêtrière, Service de Neurologie 2-Mazarin, 75013 Paris, France

## Abstract

Glioblastomas are the most frequent and aggressive primary brain tumors which are expressing various evolutions, aggressiveness, and prognosis. Thus, the 2007 World Health Organization classification based solely on the histological criteria is no longer sufficient. It should be complemented by molecular analysis for a true histomolecular classification. The new 2016 WHO classification of tumors of the central nervous system uses molecular parameters in addition to histology to reclassify these tumors and reduce the interobserver variability. The aim of this study is to determine the prevalence of* IDH* mutations and* EGFR* amplifications in the population of the northeast region of Morocco and then to compare the results with other studies.* Methods*.* IDH1 *codon 132 and* IDH2 *codon 172 were directly sequenced and the amplification of exon 20 of* EGFR* gene was investigated by qPCR in 65 glioblastoma tumors diagnosed at the University Hospital of Fez between 2010 and 2014.* Results*. The R132H* IDH1 *mutation was observed in 8 of 65 tumor samples (12.31%). No mutation of* IDH2* was detected.* EGFR* amplification was identified in 17 cases (26.15%).* Conclusion*. A systematic search of both histological and molecular markers should be requisite for a good diagnosis and a better management of glioblastomas.

## 1. Introduction

Gliomas consist of more than 80% of brain tumors. Consequently, the descriptive epidemiology of gliomas is often treated in a wider context of brain tumors. The incidence estimation of brain tumors is often difficult due to the incompleteness of the data collected and the lack of reproducibility of the current classifications.

In United States, data collection is centralized by specialized register called Cerebral Brain Tumor Registry of the United States (CBTRUS). In France, two major sources of data are available: the specialized registry of central nervous system tumors of the Gironde existing since 1999 and French Brain Tumor DataBase (FBTDB) existing since 2005. The incidence of central nervous system tumors is currently around 18/100000 in France [1–3] and 21.42/100,000 in the USA [4].

Currently in Morocco, there is a global register of Casablanca region for data collection of brain tumors. The estimated standardized incidence of central nervous system cancers is 2.0 per 100,000 men/year and 1.1 cases per 100,000 women/year [5].

In Fez region, there is not any register of cancer. However, a retrospective study tried to establish the epidemiological profile of 5532 cancer cases collected at the University Hospital of Fez between 2004 and 2010. 129 cases (2.3%) of nervous system cancer representing 10 new cases per 100,000 were reported during the same period [6].

The classification reference of gliomas was established by the World Health Organization (WHO). This classification was based on the recognition of morphological features of tumor cells compared to normal cells and malignancy grade (from I to IV). This last parameter takes into account a number of histological criteria of malignancy including cell density, nuclear atypia, mitoses, microvascular proliferation, and necrosis [7].

The 2007 WHO classification used by neuropathologists is not satisfactory. Indeed, there is great variability in the aggressiveness, prognosis, and disease progression [8]. Furthermore, there is a lack of interobserver and intraobserver reproducibility which is varying between 20 and 30% [9, 10]. Advances in molecular biology allowed establishing a histomolecular classification of gliomas and identifying more specific genetic alterations [8, 11, 12].

Glioblastoma is the most common and devastating primary brain tumor. Despite aggressive therapeutic intervention, the recurrence rate remains high with a median survival of 15 months [13, 14]. Therefore, developing new molecular biomarkers was an urgent requirement. Indeed, the identification of these biomarkers is beneficial for managing and monitoring glioblastoma patients.

The purpose of this work is to determine the prevalence of* IDH* mutations and* EGFR* amplifications in the northeast region of Morocco population. The results obtained will be integrated as a molecular approach in the diagnostic process and management of glioblastoma in Morocco. The combination of new markers with histological criteria allows establishing a histomolecular classification of these heterogeneous tumors, therefore overcoming the 2007 WHO classification limitations.

## 2. Materials and Methods

### 2.1. Patients and Samples

This is a retrospective study of 65 glioblastomas diagnosed between January 2010 and December 2014 in the Department of Anatomicopathological and Molecular Pathology, University Hospital of Fez (Morocco). Research use of tissues and anonymization of data were in accordance with local ethical approvals. The histological classification was based on the criteria set by the World Health Organization (WHO classification version 2007).

The magnetic resonance imaging (MRI) data of all patients were reviewed by a senior radiologist to confirm the diagnosis of glioblastoma.

The main criterion for inclusion of tumors in our study was the confirmation of the diagnosis of glioblastoma by neuropathologists and radiologists. Any discrepancies have led to the exclusion of the case from the study.

### 2.2. Histological and Immunohistochemical Study

Histological analysis was based on the criteria set by the World Health Organization (WHO classification version 2007). It was performed in tumor tissues that have been previously fixed in 10% formalin and embedded in paraffin. A slice thickness of 4 *μ*m was achieved then a staining was performed with hematoxylin and eosin (HE). To confirm the diagnosis and delimit the tumor areas, two different neuropathologists conducted the reading of HE slides of all patients included in the study. Then, suspicious cases underwent immunohistochemistry study using two distinct antibodies alpha-internexin (Clone RB13854 [ABGENT]) and OLIG2 (Clone EP112 [EPITOMICS]). The IHC technique was performed on a Ventana BenchMark automated according to guidelines of the manufacturer.

The alpha-internexin (INA) is a class IV neurofilament involved in neuronal morphogenesis. This is a predictive marker for 1p/19q codeletion. It is a cytoplasmic immunostaining with a paranuclear expression. Immunohistochemistry expression of INA is predictive of 1p/19q codeletion if more than 10% of tumor cells were positive with at least one tumor cluster.

The OLIG-2 is an oligodendroglial transcription factor expressed by the neural tube precursors during embryonic development and mature oligodendrocytes. Immunostaining is detected at the nuclear level.

Using these two antibodies allows for a differential diagnosis of glioblastomas and anaplastic oligodendrogliomas.

### 2.3. Molecular Study

DNA was extracted from the same tumor zones delimited by neuropathologists. The extraction was performed using QIAamp® DNA FFPE Tissue kit (Qiagen) while following the protocol described by the manufacturer. Then, qPCR was performed to study amplification of EGFR gene on Applied Biosystems™ instrument ABI 7500.

For this study, we investigated the amplification of exon 20 of* EGFR* gene. Two pairs of primers were used: Afterward Primer GTGCAGATCGCAAAGGTAATCAG and GCAGACCGCATGTGAGGAT Reverse Primer with the use of TAQMAN probe CCCCTCCCCGTATCTC.

The reaction medium is prepared from a PCR Master Mix ready for use (TaqMan® Fast Universal PCR Master Mix (2x) [Applied Biosystems]). It consists of 2x concentrated solution containing both Taq polymerase and dNTPs and all necessary components for PCR, except primers, probe, and DNA. The* EGRF* gene was amplified as follows: 2 minutes at 50°C; 15 minutes at 95°C; 40 cycles of 95°C for 15 seconds; and 60°C for 1 minute.

Besides, PCR, purification, and sequencing techniques were used to detect punctual mutations in* IDH1* and* IDH2* genes. PCR primers were used as follows:* IDH1* Forward primer 5′-AGA AGA GGG TTG AGG AGT TCA A-3′ with reverse primer 5′-CAC ATA CAA GTT GGA AAT TTC TGG-3′ and for* IDH2* 5′-TTG GCA GAC TCC AGA GCC CA-3′ with reverse primer 5′-GCC CGG TCT GCC ACA AAG TC-3′.

PCR was performed using Platinium® Taq DNA polymerase (Invitrogen). PCR conditions were 94°C for 5 minutes; 40 cycles of 94°C for 30 seconds, 60°C for 45 seconds, and 72°C for 1 minute; and extension at 72°C for 10 minutes. PCR products were purified using illustra™ ExoProStar™ 1-Step according to the manufacturer's instructions. Finally, the purified PCR products were subject of direct sequencing using the previous primers and the BigDye Terminator V3.1 Sequencing Kit (Applied Biosystems) on a 3500Dx automated sequencer (Applied Biosystems).

## 3. Results

### 3.1. Clinical Characteristics

The study included 65 cases of glioblastomas while additional 10 cases were excluded since they did not fit the inclusion criteria. The mean age of patients with glioblastoma diagnosis was 45.89 years. Patient's age ranged from 8 to 83 years, with a median value of 50 years. 55.38% of patients (36/65) were more than 40 years old while 44.62% (29/65) were young adults (≤40 years). There was a male preponderance in the studied population with male/female ratio of 1.5 (60% male and 40% female). The tumoral localizations in studied cases were 33 cases revealed in the frontal lobe, 20 in the temporal lobe, 9 in the parietal lobe, 1 in the occipital lobe, and 2 in other localizations. Most patients included in this study underwent complete tumor resection (43/65, 66.15%), while 15 patients (23.08%) had a partial resection and 7 cases (10.77%) underwent biopsy (Table 1).

### 3.2. Imaging Characteristics

**Figure 1:**
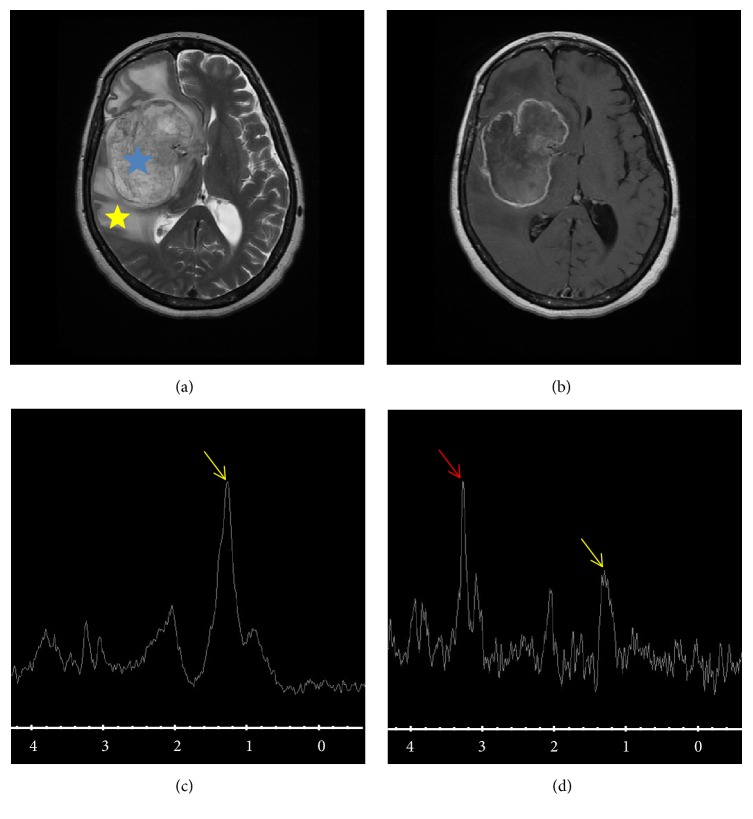
Brain MRI, T2 sequence (a), T1 weighted sequence (b), short TE spectroscopy (c), and long TE spectroscopy (d). The figure shows a voluminous brain tumor, part of our series of study, with right hemispherical localization (blue asterisk), heterogeneous and necrotic, associated with significant peritumoral edema (yellow asterisk). Choline peak (red arrow) witnesses a significant cell proliferation with lipid peak (yellow arrow) witness of necrosis. This spectroscopic and morphological appearance strongly suggests glioblastoma.

Typically, glioblastoma occurs as a large hemispherical mass with heterogeneous density and signal and important peritumoral edema. The heterogeneity is associated with necrotic component that appears on T2 and FLAIR. Intratumoral hemorrhage results in a spontaneously hyperdense appearance on CT and hyperintense T1 weighted MRI and commonly contributes to the heterogeneous presentation. The contrast enhancement is almost constant, intense, and irregular. MRI is presenting aspect of ring around the necrotic areas. Its appearance from butterfly wing side of the corpus callosum is particularly evocative when the lesion is necrotic. In MR spectroscopy is generally demonstrating a very important elevation of choline and NAA reflecting a collapse of a marked proliferation. This aspect is associated with intense signs of neoangiogenesis in perfusion MRI (Figure 1).

### 3.3. Histological and Immunohistochemistry Results

Retroreading of histological cases of glioblastomas was performed to confirm the clinical diagnosis. This allowed excluding one case from the study. Additionally, discordance between imaging and histological findings allowed ruling out three patients from the study including the case excluded by the histological study.

Histological study has identified 15 doubtful cases of glioblastoma due to disagreement of diagnosis between neuropathologists. Therefore, an immunohistochemistry study was conducted using alpha-internexin and OLIG-2 antibodies to define the histological type and to allow grading of tumors. This technique allowed confirming the diagnosis of 8 cases of glioblastomas and reclassifying, within another group, 7 remaining cases which showed a positive immunostaining of both INA and OLIG-2 antibodies. These tumors passed from glioblastoma to anaplastic oligodendroglioma group.

In total, 10 tumors were excluded; hence the final number of studied cases of glioblastoma was 65.

### 3.4. Genetic Results

Genetic study focused on identifying the status of* EGFR*,* IDH1*, and* IDH2* genes in 65 cases of glioblastoma described above.

qPCR allowed assessing amplification status of* EGFR* gene. Indeed,* EGFR* amplification was identified in 17 cases (26.15%) of glioblastomas. High-copy* EGFR* amplification (ΔCt > 5) was detected in 15/17 cases (88.24%) with* EGFR* amplification.

Exons 4 of* IDH1* and* IDH2 *genes were screened in all cases of glioblastoma targeting possible mutations. For* IDH1* gene, the punctual mutation localized at codon 132 (CGT → CAT) has been reported in 12.31% (8/65) of cases. No case of glioblastoma was detected with* IDH2* gene mutation.

## 4. Discussion

Our aim was to integrate molecular markers in glioblastoma diagnostics for better therapeutic management of patients. In fact, glioblastomas are heterogeneous tumors. Several studies have shown that cells within an individual glioblastoma are different in their morphology, genetics, and biological behavior. This is what makes the diagnosis difficult; the design of effective targeted therapy becomes challenging as well. The intratumoral heterogeneity could contribute to poor response to targeted therapy [15–17]. Histological classification based on WHO criteria, which was until recently the current classification for neuropathologists, does not explain this heterogeneity. In our Anatomic Pathology Laboratory, we noticed an interobserver discordance of 23% (15 cases). This percentage is consistent with data from the literature (20–30%) [9].

The immunohistochemistry of the 15 suspicious cases allowed confirming diagnosis of 8 glioblastomas and reclassifying 7 others as anaplastic oligodendrogliomas. Therefore, the last 7 cases were excluded from the study.

Moreover, the imaging based criteria (CT and MRI) are not sufficient to explain tumor heterogeneity as well as variability of evolution [18, 19]. In our case, there was a discrepancy between the histological and imaging analysis (3 cases). Finally, 10 cases (13.33%) were excluded from the study.

Hence, it is required to use a global approach incorporating the histological criteria and the protein expressing and genetic abnormalities that are present in glioblastoma tumors in order to decide in the diagnosis of these complex tumors and subdivide them into molecular subgroups. It would ensure a better response to treatment and a good patient outcome [20]. Recently, the new WHO classification of 2016 came to break with the old method of pathological diagnosis totally based on the morphology of tumor cells by the incorporation of certain molecular parameters in this classification. The integrated use of phenotypic and genotypic parameters in the 2016 WHO classification of brain tumors adds objectivity to the diagnostic process in the hope that this may lead to more accurate diagnosis, effective prediction of treatment response, an accurate assessment of prognosis, and therefore a good patient outcome [11, 20].

The hallmark genetic aberration in glioblastomas is mutations of* IDH* gene. This is a key tumor marker that was the basis for the restructuring and the creation of new entities in the new WHO classification version 2016.

*IDH* mutational status helps in predicting diagnosis and prognosis of glial tumors. Several studies have demonstrated that the presence of* IDH* mutations predicts significantly longer survival and progression-free survival of patients with glioblastoma [21, 22]. Consequently,* IDH* mutations constitute major alterations required to guide treatment decisions for patients with glioblastoma [23].

*IDH* mutation associated with other gene alterations impairs cellular differentiation and promotes tumor development. First-generation inhibitors of IDH mutant are in clinical trials stage and have shown so far encouraging results. Indeed, an IDH1 inhibitor (AGI-5198) has been demonstrated to bind and inhibit IDH1 mutant. Several studies tried to target protein function upstream or downstream of IDH mutant. The results would yield promising novel therapeutic strategies [24–26]. Furthermore, recent studies investigated the possibility of immunotherapeutic targeting of* IDH* mutations and suggested that IDH-targeted mutant could elicit potent antitumor immune response [27–29].

In our study,* IDH1* mutations have been identified in 8 cases among 65 studied glioblastomas (12.31%). No* IDH2* mutation was detected in the same study group. All* IDH1* mutated tumors showed* IDH1*-R132H mutation. This result is consistent with subsequent publications that have detected the same mutation in 10% and 16.8% of glioblastomas [30–32].

Univariate analysis was conducted to determine possible correlation between different clinical variables and molecular results. One statistically significant correlation was found between the mutation status of* IDH1* gene and extent of surgery (*p* = 0.03). Indeed, the majority of patients with* IDH1* mutated gene underwent total tumor resection and therefore could allow better prognosis. This result is consistent with published literature that identified* IDH1* mutation as an independent factor of good prognosis [21, 22].

Another important genetic aberration associated with glioblastoma and mentioned in the 2016 WHO classification is the amplification of epidermal growth factor receptor (EGFR) gene, also referred to as (ERBB1 or HER1).* EGFR*, localized on 7p12, encodes a membrane receptor tyrosine kinase which is bound by EGF ligand. This binding activates dimerization and autophosphorylation receptor of cytoplasmic domain. The most common* EGFR* alteration associated with glioblastoma is amplification. It was identified in up to 40% of primary glioblastomas although rarely in secondary glioblastomas. All primary glioblastomas with* EGFR *amplification showed EGFR overexpression. However, 70 to 90% of primary glioblastoma with EGFR overexpression demonstrated* EGFR *amplification [33]. Recent study has demonstrated that EGFR amplified/overexpressing glioblastomas strongly benefited from metronomic temozolomide-based therapies [34].

Besides, tumors with* EGFR* amplification might demonstrate additional alterations in the same gene. This includes in-frame deletion of exons 2–7 (*EGFRvIII*), which is present in 50 to 60% of glioblastomas with EGFR amplification. However, a recent study has demonstrated that improved survival of glioblastoma patients benefiting from metronomic temozolomide-based therapies and showing an EGFR overexpression is associated with activated EGFR/PI3K/Akt pathway independently of the presence of EGFRvIII [34].

It is therefore important to determine* EGFR* gene status for a better stratification of patients included in clinical trials but also in the case of personalized therapies [35].

In our study, 26.15% (17/65) of glioblastoma patients showed* EGFR* amplification.

Of the samples, high-copy* EGFR* amplification, as defined by ΔCt > 5 between controls and* EGFR*, was present in 15 of 17 (88.24%) samples with* EGFR* amplification. This result is consistent with previous studies where* EGFR* gene amplification frequency was, respectively, 24%, 38.3%, and 44% [36–38].

The analysis of* EGFR* and* IDH1* genes status allowed demonstrating that one case showed an amplification of the* EGFR* gene among 8 cases of* IDH1* mutated. This result is in agreement with the suggestion that* EGFR* amplification is an alteration characterizing mostly primary glioblastomas while* IDH1* gene mutations mainly concern secondary glioblastomas. Thus, 7 mutated cases were probably secondary glioblastomas and 15 cases could be primary GBM with EGFR amplified and nonmutated* IDH*. Moreover, a statistically significant correlation between (*IDH*,* EGFR*) genetic alterations and age of patients was not acquired to help a better distinction of both forms of glioblastomas. This is probably due to the small sample size. Therefore, it would be interesting to continue the study by including more cases and integrating more genetic alterations such as TP53, YKL40, and NF-1 alterations; this should allow a better classification of glioblastomas in molecular groups [20].

To date, glial tumors in Moroccan population have not been investigated, except one study involving molecular analyses of* TP53* and* IDH1/2* genes in 34 primary glioblastoma cases with no mutation identified in* IDH1/2* genes [39]. This study is complementary to the first Moroccan study and whose results are consistent with those of international literature. We identified the prevalence of common molecular markers of glioblastomas and made clinicopathological correlations with molecular data in order to identify molecular groups with good prognosis from poor or intermediate prognosis.

## 5. Conclusion

The histological classification is required for the diagnosis of glioblastoma. However, the identification of several molecular markers has become essential for refinement of glioma classification and improves prediction of treatment response and outcome. It will probably establish molecular groups with specific therapeutic algorithm for the Moroccan population.

## Figures and Tables

**Table 1:** Clinical and molecular data.

Gender	
Male	39
Female	26
Age (mean/median/range)	45.89/50/(8–83)
≤40 years	36 (55.38%)
>40 years	29 (44.62%)
KPS	
>70	60 (92.31%)
≤70	5 (7.69%)
Tumor location	
Frontal	26 (40%)
Parietal	5 (7.69%)
Temporal	8 (12.31%)
Occipital	1 (1.54%)
Two cerebral lobes affected	23 (35.38%)
Other	2 (3.08%)
Extent of surgery	
Complete resection	43 (66.15%)
Partial resection	15 (23.08%)
Biopsy	7 (10.77%)
IDH gene	
Mutant	8 (12.31%)
Wild type	57 (87.69%)
EGFR gene	
Amplified	17 (26.15%)
Not amplified	48 (73.85%)

This table summarizes the clinicopathological features and molecular results concerning our study population of 65 Moroccan patients with glioblastomas.
